# *Clostridioides difficile* Infection: A Never-Ending Challenge

**DOI:** 10.3390/jcm11144115

**Published:** 2022-07-15

**Authors:** Nicola Petrosillo

**Affiliations:** Infection Prevention and Control, Infectious Disease Unit, University Hospital Campus Bio-Medico, 00128 Rome, Italy; n.petrosillo@policlinicocampus.it

The most common worldwide cause of antibiotic-associated diarrhea/colitis is the toxin-producing bacterium *Clostridioides difficile*. After more than 80 years since the detection of this bacterium in newborn stools, and its subsequent identification as a cause of infective diarrhea, there are still difficulties in obtaining a quick and accurate diagnosis, despite the range of sensitive and specific diagnostic tools that are available; in short, the ideal diagnostic algorithm is still a matter of debate. Actually, diagnostic methods and surveillance vary across regions and countries, hampering a global and more precise overview of the burden of *Clostridioides difficile* infection.

During the last decades, major changes occurred in diagnostic methods for the detection of toxigenic *C. difficile*; direct toxin enzyme immunoassay (EIA), antigen glutamate dehydrogenase (GDH) have been the diagnostic standard for years with problems of sensitivity and specificity and, partly, of turn-around time. More recently, molecular tests have entered the clinical-diagnostic practice. Tests such as nucleic acid amplification assays (NAAT) are fast and more sensitive diagnostic assays and probably contributed to a substantial increase in the reported incidence of *C. difficile*. NAAT’s high sensitivity, however, could lead to an overdiagnosis of CDI, especially in carriers of toxigenic strains. The unresolved question of the clinical significance of the role of the asymptomatic carriage of a toxigenic strain therefore makes this point an unmet need.

Another point to be addressed is the potential role of diagnostics to predict the clinical outcome of patients with *C. difficile* infection, principally the risk of recurrence. Quantitative NAAT could have the potential to provide toxin quantification in the stool, which could be used as a marker of infection severity and clinical outcomes. In a very comprehensive review in this Special Issue, Kampouri E. et al. [1] highlighted the main unmet needs in the diagnosis of *Clostridioides difficile* infection. While diagnosis cannot be achieved with a single assay, the use of NAAT as a standalone test in high *C. difficile* prevalence conditions is promising and needs further studies. Moreover, more population-based studies are needed to better evaluate the true burden of *C. difficile* infection in the community and its associated risk factors. Findings from these studies could increase general practitioners’ awareness on the diagnosis of *C. difficile* in the community and provide them with specific recommendations for testing for *C. difficile* in patients with diarrhea outside the hospital setting.

The occurrence of the *Coronavirus* disease 2019 (COVID-19) pandemic is certainly not helping us to address the unmet needs of *C. difficile* infection. This pandemic has somewhat upset most certainties in public health and in clinical practice. Due to the high burden of symptomatic patients with severe acute respiratory syndrome coronavirus 2 (SARS-CoV-2), COVID-19 has introduced disruption in all healthcare services and increasing levels of healthcare resource utilisation, with most countries reporting one or more disruptions to essential health services. The ongoing surveillance of many infections, including those caused by multidrug-resistant organisms, tuberculosis, malaria, human immunodeficiency virus (HIV), and vaccination-preventable diseases, has been disrupted or strongly reduced. This pandemic has also taken a substantial toll on academic progress and on the training of healthcare providers. Many young doctors in training had their training interrupted and were abruptly launched in the agon of the COVID-19 wards.

In addition, antimicrobial stewardship programs have skipped in several institutions, just when 70% and more of COVID-19 patients were given antimicrobials without any rationale and contrary to international and national recommendations.

In the hottest periods of the COVID-19 pandemic, in the hospitals overflowing with patients with SARS-CoV-2 infection, attention towards other infectious diseases decreased. *C. difficile* infection was one of those that paid the price, with fewer diarrheal stool samples sent to the laboratory and with a reduction in the incidence of laboratory *C. difficile* infection diagnoses.

What has been the impact of COVID-19 on *C. difficile* infection? In one of the widest studies of *C. difficile* burden in COVID-19 patients, from February through July 2020, 8402 COVID-19 patients admitted to eight Italian hospitals were studied [2]. Among them, 38 *C. difficile* infection cases were identified, of which 32 were hospital-onset, with an incidence of 4.4 cases × 10,000 patient-days. The presence of a previous hospitalization (*p* = 0.001), previous steroid administration (*p* = 0.008) and the administration of antibiotics during the stay (*p* = 0.004) were risk factors associated with *C. difficile* infection. These data are consistent with those produced before the pandemic, with the difference that, in most COVID-19 patients, antimicrobials are unnecessary.

As the dust from the COVID-19 pandemic settles, we will need even more solid epidemiological data on morbidity, mortality, and risk factors to tackle relevant infectious diseases such as *C. difficile* infection. In this Special Issue, Granata G. et al. [3] illustrate the findings from a prospective incidence study carried out in 15 Italian hospitals, in which 271 patients with *C. difficile* infection were followed-up for 30 days, for a total of 7795 patient-days. Firstly, the crude all-cause mortality rate at 30 days was 10.7% (33 deaths/309 patients). Moreover, recurrence of *C. difficile* infection occurred in 21% of the followed-up patients at 30 days, with an incidence rate of 72 recurrences/10,000 patient-days. Logistic regression analysis identified exposure to cephalosporin as an independent risk factor associated with recurrent CDI (*p* = 0.03). Of note, among the 12 patients in which fidaxomicin was given at the first *C. difficile* infection episode, no one experienced a recurrence; these data confirm the role of fidaxomicin in preventing recurrence, as recently stated by US and European guidelines that recommend fidaxomicin as first choice treatment for the first *C. difficile* infection episode, especially for patients at high risk of recurrence [4,5].

The high rates of infection recurrence mean that treatment strategies are constantly under review. Recurrences indeed remain the main challenge in the clinical management of patients with *Clostridioides difficile* infection; the recent updated international guidelines also definitively allowed entry of monoclonal antibodies against B toxin, Bezlotoxumab, in the prevention of recurrences. A real-word experience on the use of Bezlotoxumab has been reported by Escudero-Sánchez et al. [6] in this Special Issue. In their retrospective, multicentre cohort study including 91 consecutive patients receiving bezlotoxumab between 2018 and 2019 in 13 Spanish hospitals, the rate of recurrence was 14.3%, lower than that reported in the literature for patients not treated with bezlotoxumab; moreover, the results from this real-word study are remarkable considering the fact that bezlotoxumab is generally used in a much more compromised population than that represented in the clinical trials that assessed the effectiveness of bezlotoxumab.

The pathogenesis of *C. difficile* infection is far from being fully elucidated; the role of toxin A and of the binary toxin are still under study [7]. The role of the humoral immune response to *Clostridium difficile* in modulating the severity of *C. difficile* infection remains unclear. In this Special Issue, Na’amnih W. et al. found higher IgG levels against TcdA and TcdB in patients with mild versus severe CDI [8]. These preliminary findings provide a basis for future large-scale studies and support the development and evaluation of active and passive immunotherapies for *C. difficile* infection management.

Finally, areas of uncertainty persist regarding the significance of asymptomatic *C. difficile* carriage, its role in the *C. difficile* transmission in the community and healthcare settings, and the potential for the re-emergence of *C. difficile* infection in peculiar clinical conditions and specific healthcare settings. Even though screening for *C. difficile* is not recommended by the main guidelines, evidences are accumulating on the role of screening for colonization in selected settings and selected patients at admission to prevent healthcare associated *C. difficile* infection, and its spread in the healthcare environment.

There is still a lot to know about *Clostridioides difficile* infection; there is not a sector of this disease that does not require more in-depth knowledge and solid pathophysiologic data. In short, it is a never-ending challenge.

## References

[1] Kampouri E., Croxatto A., Prod’hom G., Guery B. (2021). Clostridioides difficile Infection, Still a Long Way to Go. J. Clin. Med..

[2] Granata G., Bartoloni A., Codeluppi M., Contadini I., Cristini F., Fantoni M., Ferraresi A., Fornabaio C., Grasselli S., Lagi F. (2020). The Burden of Clostridioides Difficile Infection during the COVID-19 Pandemic: A Retrospective Case-Control Study in Italian Hospitals (CloVid). J. Clin. Med..

[3] Granata G., Petrosillo N., Adamoli L., Bartoletti M., Bartoloni A., Basile G., Bassetti M., Bonfanti P., Borromeo R., Ceccarelli G. (2021). Prospective Study on Incidence, Risk Factors and Outcome of Recurrent Clostridioides difficile Infections. J. Clin. Med..

[4] Johnson S., Lavergne V., Skinner A.M., Gonzales-Luna A.J., Garey K.W., Kelly C.P., Wilcox M.H. (2021). Clinical Practice Guideline by the Infectious Diseases Society of America (IDSA) and Society for Healthcare Epidemiology of America (SHEA): 2021 Focused Update Guidelines on Management of Clostridioides difficile Infection in Adults. Clin. Infect. Dis..

[5] Van Prehn J., Reigadas E., Vogelzang E.H., Bouza E., Hristea A., Guery B., Krutova M., Norén T., Allerberger F., Coia J.E. (2021). European Society of Clinical Microbiology and Infectious Diseases: 2021 update on the treatment guidance document for Clostridioides difficile infection in adults. Clin. Microbiol. Infect..

[6] Escudero-Sánchez R., Ruíz-Ruizgómez M., Fernández-Fradejas J., García Fernández S., Olmedo Samperio M., Cano Yuste A., Valencia Alijo A., Díaz-Pollán B., Rodríguez Hernández M.J., Merino De Lucas E. (2020). Real-World Experience with Bezlotoxumab for Prevention of Recurrence of Clostridioides difficile Infection. J. Clin. Med..

[7] Rohana H., Azrad M., Nitzan O., Adler A., Binyamin D., Koren O., Peretz A. (2020). Characterization of Clostridioides difficile Strains, the Disease Severity, and the Microbial Changes They Induce. J. Clin. Med..

[8] Na’amnih W., Carmeli Y., Asato V., Goren S., Adler A., Cohen D., Muhsen K. (2020). Enhanced Humoral Immune Responses against Toxin A and B of Clostridium difficile is Associated with a Milder Disease Manifestation. J. Clin. Med..

